# Mr. Jones's Case of Puerperal Convulsions

**Published:** 1813-12

**Authors:** William Jones

**Affiliations:** Marlborough


*To the Editor of the Medical and Physical Journal.*

SIR,

ALTHOUGH there may be nothing novel in the symptoms or treatment of the following case of **true* Puerperal Convulsions; yet, as it belongs to a formidable class of diseases whose termination is too generally fatal in proportion with their occurrence (which, however, is providentially not frequent); and as this case terminated favorably, though attended from the onset with most alarming symptoms; it will, I trust, be readily recorded in your respectable Journal, as it may tend, perhaps, to encourage *perseverance*, and perpetuate the motto of *Nil desperandum.*

I am, Sir,
Yours respectfully,
WILLIAM JONES.

*Marlborough,*
*October* 18, 1813.

On the 23d of September last, about ten A. M. I was desired to visit A. B., an athletic woman, æt. 24, the wife of a laboring man, who, I was informed, had that morning, soon after rising, been attacked with convulsion fits, though not before subject to them. I found her sitting in a chair in a state of stupor, from which having roused her by the use of volatile stimuli, &c. I put several questions, which she answered rationally but rather incoherently; and complained of pain only in the head, with giddiness and indistinct vision; nausea, with inclination to vomit; and the pupil of the eye was also somewhat dilated. Not having been previously apprised of her being pregnant, I inquired, on observing the *abdomen rotundum*, how far she was advanced in her gestation, and learnt it was nearly or quite completed, with her first child.

There was at this time no apparent symptom of the commencement of labour, except a slight rigor, which, though a frequent attendant on the first dilatation of the os uteri, might possibly in this case have been attributed to the convulsions. The pulse was rather languid and slow. Being suspicious, however, that these unpleasant symptoms were the *precursors* of labour, I abstracted about twenty ounces of blood from the arm, and desired she might be kept quiet and recumbent, at the same time intimating to her attendants

* The reporter is induced to make this distinction in consequence of observing, in No. 173 of Med. and Phys. Journal, p. 36, a case of Convulsions during Labour, which, however, he trusts no reflecting or discerning practitioner would consider as a case of *true* puerperal convulsions.

ants that labour would probably come on ere long; and desired to be sent for immediately on the accession of pains, or other signs of labour. About eight P. M. one of the attendants informed me she considered her to be in labor, though the patient appeared quite insensible of any such event; and on examination (per vaginam) I found the os uteri pretty fully dilated, and the head of the child, with the membranes entire, about to pass into the pelvis. She was laboring under a violent convulsive struggle, the uterus also apparently acting strong. I waited the continuance of two or three pains, and observed the convulsions (which were now so strong that she was with difficulty kept on the bed by the aid of four women) to be immediately excited by, or at least simultaneous with, each expulsive effort of the uterus.

Aware of the attendant danger and too often fatal termination of similar cases, I called in a more experienced practitioner (Mr. W. of this place), when it was agreed to proceed to immediate delivery; and, although there are different opinions on the propriety of proceeding to deliver in such cases, I trust it will not be questioned in this instance, as the child's head had descended favorable for the application of the forceps, and a full-grown living male child was readily extracted in about twenty minutes. The convulsions now temporarily subsided, and she lay in a state of coma, with stertorous respiration, until the action of the uterus recommenced for the expulsion of the placenta, which was effected within a quarter of an hour from the birth of the child, when, during the action of the uterus for its detachment, the convulsions were again excited; after which she relapsed into the comatose state, and appeared totally insensible of the delivery of the child or placenta.

A draught with sixty drops of T. Opii was directed to be taken immediately; and afterwards, when a favorable opportunity offered, two or three table-spoonfuls of a mixture with Sp. Ammon. c. Æth. Vitr. c. Lav. c. et Mist. Camph. but owing to a difficulty of swallowing the draught was wasted, and only two table-spoonfuls of the mixture were taken during the night, which was passed restlessly, and accompanied with several convulsion fits.

On the morning of the 24th I found her in a strong convulsion; her lips black; respiration short, difficult, and occasionally suspended; pupil of the eye insensible to the light of a candle; pulse small and irregular, but after the subsidence of the fit full and quick. Venesection was proposed, but her relatives objected to it, as they considered her in a dying state. Sinapisms were applied to the feet, and an enema enema ordered to be administered. On visiting her again at noon, and finding the pulse still full and quick, I insisted on the necessity of abstracting more blood, which I now did from the arm to about twenty-four ounces, which was buffy. A blister was applied to each leg. From this time till eight in the evening she had only one strong convulsive fit. Two unsuccessful attempts having been made to throw up the injection, it was ordered to be repeated, with the addition of Sulphas Magnesiæ ℥ij. A diaphoretic draught was given, which produced some determination to the skin.

The night was passed quiet without any strong convulsion, and on the morning of the 25th there appeared a ray of returning sensibility, as she now opened her eyes on being called by her name, and made an effort to open the mouth on some gruel being offered. The pulse was softer, but quick. The enema had not succeeded, and two or three table spoonfuls of an aperient mixture were directed to be given whenever a favorable occasion presented, and a large blister was applied *inter scapulas.*

The night was passed quiet, and she was better on the morning of the 26th; but as little of the mixture had been taken, it was ordered to be continued, and the enema repeated, which soon after produced a copious alvine evacuation, and discharge of urine, to both of which functions she was now sensible, though her urine had hitherto passed involuntarily. The skin was somewhat moist; pulse soft, and not so quick. She had now *risus sardonicus,* laughing immoderately at any one who approached the bed, though still unable to speak.

The night was passed quiet, but sleepless; and she appeared still better on the morning of the 27th. The countenance was more natural, and she spoke for the first time since the commencement of her labour, but of which, with concomitant circumstances, she has of course no recollection, though she now answers any interrogatories (unconnected therewith) rationally and promptly, but with an unusual quickness. The secretion of milk being considerable, the breasts were drawn, as she would not acknowledge the child, or suffer it to remain sufficiently long at the breast to be useful.

The night was passed quiet, though sleepless; and she appeared much amended on the morning of the 28th. The pulse was regular and soft; had a natural evacuation from the bowels; the skin continued moist; and she now admitted the child to suck with seeming parental solicitude. Thirty drops of Tinct. Opii were given at bed-time, which procured

 four four hours sleep towards the morning of the 29th, by which she was much refreshed. She now took sufficient liquid nutriment, and having had no convulsion since the 24th, was considered convalescent; and on seeing her upon the 3d of the present month, I found her quite recovered.

